# The Potential of Using Temperate–Tropical Crossbreds and Agricultural by-Products, Associated with Heat Stress Management for Dairy Production in the Tropics: A Review

**DOI:** 10.3390/ani12010001

**Published:** 2021-12-21

**Authors:** Predith Michael, Clement Roy de Cruz, Norhariani Mohd Nor, Saadiah Jamli, Yong Meng Goh

**Affiliations:** 1 Institute of Tropical Agriculture and Food Security, Universiti Putra Malaysia (UPM), Serdang 43400, Selangor, Malaysia; predith77@gmail.com; 2 Livestock Science Research Centre, Malaysian Agricultural Research and Development Institute Headquarters, Persiaran MARDI-UPM, Serdang 43400, Selangor, Malaysia; saadiahj@mardi.gov.my; 3 Department of Aquaculture, Faculty of Agriculture, Universiti Putra Malaysia (UPM), Serdang 43400, Selangor, Malaysia; decruz.cr@upm.edu.my; 4 Department of Veterinary Preclinical Sciences, Faculty of Veterinary Medicine, Universiti Putra Malaysia (UPM), Serdang 43400, Selangor, Malaysia; norhariani@upm.edu.my

**Keywords:** dairy production, tropic, crossbred, agriculture by-product, heat stress

## Abstract

**Simple Summary:**

The two main factors projected to boost milk production are genomic selection and improvement in the quality and digestibility of feedstuff. The focus of the current article is to look at available measures to curb heat stress and enhance the dairy productivity of tropical crossbred cattle, using readily available agricultural by-products in tropical regions. Inherent associations explored by the current work among breed attributes, agricultural by-products, feeding efficiency in the challenging tropical climate, and heat stress management would be invaluable in constructing a predictive and decision support system for farmers.

**Abstract:**

The demand and consumption of dairy products are expected to increase exponentially in developing countries, particularly in tropical regions. However, the intensification of dairy production to meet this increasing demand has its challenges. The challenges ranged from feed costs, resources, and their utilization, as well as the heat stress associated with rearing temperate–tropical crossbred cattle in the tropics. This article focused on key nutritional and environmental factors that should be considered when temperate–tropical crossbred cattle are used in the tropics. The article also describes measures to enhance the utilization of regional feed resources and efforts to overcome the impacts of heat stress. Heat stress is a major challenge in tropical dairy farming, as it leads to poor production, despite the genetic gains made through crossbreeding of high production temperate cattle with hardy tropical animals. The dependence on imported feed and animal-man competition for the same feed resources has escalated feed cost and food security concerns. The utilization of agricultural by-products and production of stable tropical crossbreds will be an asset to tropical countries in the future, more so when scarcity of feed resources and global warming becomes a closer reality. This initiative has far-reaching impacts in the tropics and increasingly warmer areas of traditional dairying regions in the future.

## 1. Introduction

Milk and dairy products are an increasingly important pillar for food security in many countries. By 2050, it is foreseen that developing countries will have 61% of world milk production, many of which are in the tropical region [1]. The global human population is predicted to be about 9–10 billion, with accelerating urbanization in most developing countries by 2050 [2,3]. The emergence of the middle-income class with better purchasing power would increase demand for dairy products [4]. Per capita, milk consumption is expected to increase from 55 kg in 2015 to 78 kg in 2050 in developing countries alone [1]. Over the years, the growth in demand for milk has been exponential. China has experienced a tripling of milk consumption per capita from 1985 to 2000, and it doubled in 2004 [5]. In India, the per capita availability of milk reached 281 gm per day in 2012. India is projected to increase its total share of world milk production from 15% to 21% by 2050 [1,6]. Milk output in Asia has increased by 2.9% from 2018 to 2019, contributing to approximately 42.2% of global milk production [7]. The African continent has recorded increased milk production by 0.3% between 2018 to 2019. These contributed to about 5.5% of global milk production, with milk consumption in the sub-Saharan regions to triple by 2050 [7,8]. Some of the main contributing factors to this are improved milk collection and processing, increased efficiency in large-scale farming, improved feeding management, and the adoption of artificial reproduction methods [7]. The increase in expected production would be led by an expected additional demand of an estimated 600 billion kilograms of milk, more in 2067 than that produced in 2018 [9].

In order to meet demand, high-yield temperate dairying breeds have been introduced to the tropics to improve dairy production in intensive systems [10]. Temperate breeds, such as Holstein–Friesian and Jersey × Holstein–Friesian, kept in a climate-controlled environments, have achieved a total milk yield of 9053 and 7438 kg per lactation, respectively [11]. In contrast, imported pure breeds, such as Friesian, could only produce up to 6570 kg in the tropics [12]. The temperate breed requires more expensive investments in good nutrition, housing, management, and heat stress mitigation systems, in order to perform in the tropics [13]. The farming conditions in the tropics highlights the need for resilient dairy breeds to withstand these management conditions. Several attempts of crossbreeding of temperate breeds with hardy indigenous tropical breeds had been undertaken in the past. Research has harnessed the desirable traits of two or more (temperate or tropical) breeds for better productive performances, while improving the adaptability of temperate–tropical crossbreds to the tropical environment [14]. For example, some of the coveted and adaptable characteristics, seen in the Sanga and Zebu (Sahiwal) cattle breeds, such as resistance to tick and tick-borne diseases [15,16], are retained in the temperate–tropical crossbreds. Other adaptable traits to mitigate heat stress are observed through changes in hair coat characteristics [17].

However, to optimize the breed performance for maximum production, sufficient nutrients need to be supplied. For example, a Jersey × Holstein–Friesian cow requires a daily dry matter intake of 12.6–14.8 kg to produce 7438 kg of milk per lactation season [11]. Achieving this in the tropical regions requires large tracts of land and amounts of high-quality feed materials, which are often imported. FAO’s feed assessment found that the growth in livestock production has propelled an increased demand for grains [5]. Due to the dependence on imported feed, the increased feed cost may further impact small and mid-scale farmers [18]. In addition, increasingly frequent and unpredictable drought conditions, associated with global warming, may further impact feed resources and escalate feed cost [19]. Therefore, there is an urgent need to increase further the utilization of agricultural residues in tropical regions [20]. More so, towards the year 2067, it is predicted that lesser land will be available for agriculture, leading to more land being used to plant human food than animal feed [9]. In tandem with the increased plantation of human food, the availability of these agricultural by-products is also expected to increase. However, it’s utilization is still less than ideal, due to the lower nutritive values than imported feed and anti-nutritive factors, but are expected to be overcome with better processing methods and developmental strategies [21]. The strategic usage of these regional feedstuffs through ration balancing could reduce the impact of heat stress, methane emission, provide sufficient nutrients, and reduce feed cost by capping on the indigenous breeds’ ability to digest low-quality roughages [18,20,22,23,24]. Unless climate and nutrition challenges are addressed, losses in milk yield can reach up to 0.96 kg/day, with a depressed dry matter intake (DMI) of 0.85 kg, when dairy cattle are subjected to heat stress [22,25].

Globally, more than 925 million smallholders who depend on livestock activities as a source for food and income are primarily found in the sub-Saharan Africa and South Asia regions [4,6]. Despite Asia producing almost 3-fold more milk yield than North America, the yield per cow is much lower [7]. Britt et al. [9] noted that 46 million cows in India produce an average of 1446 kg per cow, per year, compared to 9.2 million cows in the USA that produce on average 10,150 kg per cow, per year. The disproportion in production would further leave a strain on farmland and feed resource availability. Milk production has also been a crucial income for many smallholders in tropical regions. In Kenya, for example, dairy cattle production contributes up to 14% of the country’s GDP, and livestock can amount to almost half of the rural household income [4,26]. The use of food material for animal feed raises a concern for food security in many developing countries. People living in poverty (below the household income level of 2 USD or less per day) depend predominantly on cereals, roots, and tubers as their staple food [1,8]. The estimated world feed use of cereals was at 742 million tons, or 36% of total world cereal use, and this is forecasted to reach above 1 billion tons by 2050 [1]. Root crops are also a principal food source for some countries in sub-Saharan Africa, with potatoes (11%), sweet potatoes (30%), and cassava (34%) of the total world production being used for animal feed [1]. Moreover, by 2030, prices of maize, wheat, sorghum, sweet potato, and oil grains are expected to double, leaving these groups of people vulnerable, due to the expansion of the livestock industry [1,27]. Therefore, to achieve sustainable dairy farming in developing countries in the tropics, challenges associated with breed performances, scarcity of feed resources, and heat stress conditions need to be overcome.

The two main factors projected to boost milk production in the future are genomic selection and improvement in the quality and digestibility of feedstuff [9]. Demand for dairy products is prevalent and expected to increase in the future. Efforts have been made to manage dairy farming in tropical conditions by using agricultural by-products in ration balancing and crossbreeding endeavors. The limitation of indigenous dairy cattle breeds’ productive ability and the adaptability challenges of the imported temperate breeds had led to crossbreeding in the tropical regions. On the other hand, the impact of climate change is expected to further restrict future feed resources and dairy production in the tropics. We aim to present an overview of crossbreeding efforts in countries along the equatorial line, mainly developing countries, while addressing some of the challenges in feed resources and heat stress in meeting future dairy demand. For the current review, developing countries are described based on countries listed by Alexandratos and Bruinsma [1], and equatorial climate are regions denoted with Af to Aw in the Köppen–Geiger climate classification [28].

## 2. Temperate–Tropical Crossbreeding Endeavors in Developing Countries in the Tropics

One of the main challenges faced by many dairy operators in the tropic is selecting a suitable dairy cattle breed adjusted to tropical conditions. Traits such as heat tolerance, adaptability towards seasonal changes, and scarcity of feed, as well as tolerance towards tick infestation and metabolic diseases, are desirable in the tropics [29]. These traits are commonly observed in indigenous dairy breeds in tropical regions, which incidentally have poor milk yield [13]. To overcome this problem, crossbreeding between temperate and tropical breeds has been carried in tropical regions such as South America, West Africa, and some regions in Southeast Asia in the early 19th and 20th centuries [30]. Temperate–tropical crossbreds tend to have better productive performances than their indigenous breed [10]. It was observed to be a faster way to produce results by significantly improving indigenous breeds [30]. Terminal and rotational crosses have also been employed to optimize the crossbred’s heterozygosity [31]. Kahi et al. [32] compared crossbreeding of first crosses, 2–3 breed rotation, and synthetic breeding and concluded that the rotational and synthetic breeding performances were close to the first crosses. The author stressed that the cost of maintaining a pure line breed with high genetic potentials for first crosses as herds or through in vitro fertilization might not be sustainable for smallholder farmers in the tropic. Rotational crossing allows larger heterosis leading to better performances but requires a well-organized farm with more meticulous efforts in recording and managing the different breeds of herds [33]. Synthetic breeds are easier to be managed and may be more suitable for smallholders’, overcoming problems faced through first crosses and rotational crosses [32]. Synthetic breeds require proper selection of inter se breeding to retain the crossbreed’s level of hybrid vigor [34]. Although synthetic breeds were reported to have maximum recombination losses, the increased genetic variation may improve selection response, possibly even superior to the parental breeds [32,35].

Several first crosses and synthetic tropical crossbreds have been developed in the past. Developing countries are referred to Alexandratos and Bruinsma [1], which listed several countries in Latin America and the Caribbean, sub-Saharan Africa, Near East/North Africa, South Asia, and East Asia. Published data were collected from regions described with equatorial climates denoted as Af (Equatorial rainforest, fully humid), Am (Equatorial monsoon), As (Equatorial savannah with dry summer), and Aw (equatorial savannah with dry winter) according to the Köppen–Geiger climate classification from the year 1996 to 2020 [28]. It was reported in South East Asia, Australian Frisian × Sahiwal (3rd generation) can produce a total milk yield per lactation of 1978 kg [36], Thai native × Frisian of 50% and 75% Frisian yielded 2009 kg and 2516 kg, respectively [12] and Holstein–Friesian × Vietnamese local yellow cattle produced 1168 kg yield [37]. In Latin America, Holstein × Gyr of 50%, 37.5%, and 87.5% Holstein was observed to produce 5118 kg, 4569 kg and, 5211 kg of milk yield, respectively [38]. In South Asia, it was found that Holstein–Friesian × Sahiwal and Jersey × Red Sindhi yielded 2381 kg and 2676 kg of milk, respectively [39,40]. In East Africa, Ethiopian Boran × Holstein of 50% and 75% Holstein produced 1831 kg and 1940 kg of milk yield, respectively [41].

It is also notable that milk production of the various temperate–tropical crossbreds (indigenous × temperate), were found to be between half to 1/3, of that of the temperate, pure breed performance [11,42], even with improved feeding rations. Most tropical crossbred produced between 2000–2500 kg/ lactation in various tropical regions. A meta-analysis study on temperate–tropical crossbreds demonstrated lower energy efficiency than temperate breeds [43]. The difference in energy efficiency could be due to the smaller organ sizes, observed in the crossbreds, compared to the temperate breed. For instance, Red Sindhi × Jersey crosses had reduced intestinal tract length and total stomach weights of 12.6% and 21.4%, respectively. Increasing the Red Sindhi bloodline in these crosses would also decrease feed intake up to 14.5%. The smaller digestive tract in crossbreds would lead to lesser feed intake and possibly reduced feed efficiency [39]. The lower feed efficiency may explain the decreased production figures observed in crossbreds, compared to temperate breeds. Despite this, crossbreeding remains a relevant strategy to improve dairy cattle production in the tropics, as housing costs to maintaining these temperate breeds under tropical conditions can be prohibitively high. For example, the optimum performance of the Israeli Holsteins could only be achieved with sufficient cooling. This comprised of 30 sprinklers (at 720 L capacity), 3 large fans (120,000 m^2^ air/h/fan), and 4 small fans (8800 m^2^ air/h/fan) [44].

The genetic value of indigenous breeds has been often overlooked. Sahiwal cows from South Asia and East Africa have been observed to yield a total milk yield per lactation of 1277 kg and 1399 kg, respectively [39,45]. Red Sindhi breed from South Asia produced 2369 kg [46], whereas, in East Africa, the Ethiopian Boran breed yielded 582 kg of milk yield per lactation [41]. In Latin America, the Brazilian Gyr and Guzerat breeds have produced 4256 kg and 1783 kg, respectively [38,47], and in sub-Saharan Africa, the Butana breed yielded 1662 kg of milk yield [48]. Most indigenous breeds can yield between 1200 kg to 2000 kg per lactation in the various tropical regions. Generally, crossbreeding with temperate breeds could improve indigenous dairy breed performance through good breeding programs and genomic selection. Selection criteria should be based on an optimum index of both lactational yield and length [49]. A summary of 23 studies on tropical crossbreds with 50% *Bos taurus* bloodlines showed an increase in milk yield and lactation length by 2.4 and 1.2 times, respectively, compared to the indigenous breeds [14]. Another study demonstrated a higher feed efficiency with a greater milk yield response to feed quality in the Holstein × Boran crossbred, compared to the indigenous Boran breed [10]. There have been numerous studies of genomic markers for selected traits that could improve the performances of crossbred dairy cattle [9]. Moreover, crossbreeding utilizes the adaptability of the indigenous breeds to the regional environments and feed and serves to preserve its genetic attributes and diversity. Ilatsia et al. [45] stressed that the lack of selection could be observed, given the sizeable coefficients of variation, leading to poorer performances of the Sahiwal breed. The exceptionally high performances of the Brazilian indigenous Gyr breed suggests that, with proper selection criteria for breeding, performances of these indigenous breeds could be further improved. The continuous importation of high-performing temperate breeds may lead to the extinction of indigenous tropical breeds. Extinction is inevitable, as these poor-performing indigenous animals are no longer valued for their production functions. From 2004 to 2015, the risk of extinction of livestock breeds increased from 15 to 17% [50]. Therefore, there is an urgent need to conserve indigenous breeds for future crossbreeding and genetic improvement.

Backcrossing has been seen as part of the breeding strategies to improve crossbreds’ performance [31]. Backcrossing in the various crossbreds has shown some potential in improving the milk yield. It was reported that four generations of backcrossing Damascus × Frisian cow led to an increase of 350-day lactation yield by 5.5%, reaching up to 4085 kg [13]. Furthermore, dairy production in Thailand, of eight different crossbreds, showed that breeds with higher Holstein genetic representation produced higher milk yield [51]. Similarly, backcross of crossbred (Holstein × Boran) of 87.5% Holstein–Friesian (HF) observed up to 15% increase in milk yield, compared to crossbred of 50% HF [41]. Backcrossing can be done to improve hybrid vigor through selected traits, such as milk yield, before establishing a blood level of the synthetic breed. Nevertheless, caution should be applied to repeated backcrossing, as, although it may mildly increase milk yield or even decline, it may also lead to decreased fertility, poorer adaptation to the tropical climate, and even mortality, in some instances [33]. McDowell et al. [39] observed declining milk performances of 1/2 H and 3/8 H of Holstein × Sahiwal that could be attributed to the indigenous breed influence and interaction effects of genotype by the environment, which may limit 5/8 H and 3/4 H crosses. Once a blood level is determined, inter se breeding, based on productive performance and adaptability to their regional climate and feed resources, can be considered as selection criterions. Paim et al. [52] suggested at least five generations of inter se breeding before achieving a stable hybrid or synthetic breed for increased variability among the hybrid progeny and adaptability to regional climate.

One of the reasons for the lowered production in the crossbreds could be the lack of funds to sustain long-term breeding programs [30]. Synthetic crossbreeding requires several generations of inter se breeding before a stable breed can be observed [31]. For example, Australian Friesian Sahiwal (AFS) took roughly 40 years of extensive breeding, at an estimated cost of AUD 30 million [14]. Long-term breeding plans and sufficient expertise in the field are deemed necessary to attain successful crossbreds [30]. Without long-term and coordinated breeding plans, crossbreds were observed to have acquired non-productive parental genes. For example, Holstein crossbreds for some dairy operators in the tropics can comprise of several component breeds. In Southeast Asia, Holstein crossbreds were observed to have parental genes of Holstein, Brahman, Jersey, Red Dane, Red Sindhi, Sahiwal, and Thai Native breeds [51]. In East Africa, Kenyan Frisian was observed to have Holstein, Norwegian Red, and Guernsey genes [53]. Breeding programs need to cater to regional needs to achieve realistic short and long-term goals, through strategic cooperation between governmental and private organizations [30]. Therefore, it is vital for breeding strategies to incorporate the ecological and socio-economic circumstances of the tropical region to attain the desired results [32]. We concur with Madalena et al. [30] and Kahi et al. [32] that continuous study on a stable synthetic or composite breed development may be more achievable for developing countries in the tropical region, based on previous successes.

Demand for established synthetic crossbreds is prevalent in many tropical countries. The Australian Frisian Sahiwal (AFS) breed is well-adapted to the tropical climate and has been exported to Mexico, Brunei, Thailand, India, and Malaysia [14]. The breed’s performance in Southeast Asian countries ranges from 1710 to 1978 kg of milk/lactation [36,54]. Another well-established breed is the Holstein × Gyr breed, where several requirement and feed efficiency studies have been conducted in recent years [55,56,57]. The breed was observed to produce an average milk yield of 5118 and 5211 kg/lactation of 1/2 and 7/8 Holstein composition, respectively [38]. Understanding genotype and environmental interactions are fundamental to optimizing dairy production in the tropic [58]. As a general guide, 25% to 75% of the genetic makeup of the indigenous breed adapted to local feed and environment is required in a crossbred animal. The genetic makeup would depend on the harshness of the region where the crossbred is to be introduced [59]. Ultimately, the success of a crossbred is determined by the access to adequate stock, the breed’s ability to express its full genetic potential, efficiency in the market chain, and the adoption of appropriate breeding strategies [31].

## 3. Managing Feed Resources

### 3.1. Strategic Manipulation of Agricultural by Product

Most of the agricultural expansion in the 21st century is expected to be centered in South America and sub-Saharan Africa, due to the large land areas with unexploited agricultural potentials [60]. The by-products of this expansion would provide a much-needed feed supply for the growing dairy industry in the tropics. These include residues from plant-based oil, distilleries and breweries, grain and legume milling, fruit and vegetable processing, sugar, starch, and confectionery industry, as well as other crop waste and residues [20]. Oil crops, such as oil palm, soybeans, and rapeseed, provide 82% of the total increment in world oil crop production. Availability of oil crop by-products for animal feed is expected to increase in tandem with the growth in biodiesel adoption, which would remain a primary source of animal feed [1]. For example, in Southeast Asian regions, such as Malaysia and Indonesia, the mass production of oil palm provides essential feed for dairy farming [61]. Grain usage for ethanol production amounted to 4.2% of the total global grain supply in 2010 [61]. Predominantly, maize has been used for ethanol production from cereal grain, but it could also be produced from wheat, barley, sorghum, and, to a lesser extent, rye, triticale, sorghum, and oats [61]. Improved processing methods used have been observed to increase the quality of the feedstuff. For instance, crude maize oil, extracted from condensed distillers soluble (CDS), was observed to mildly increase protein and reduce fat content in distillers dried grain (DDGS) [61].

New manipulation strategies in improving the utilization of agricultural by-products would increase its usage in ration balancing for dairy cattle diets. Continuous assessment of these by-products’ availability, digestibility, toxic profiles, and nutritive value is essential to ensure sufficient nutrient supply for dairy production [5,20,61]. Several methods have been proposed to improve its utilization. These included physical, chemical, biological, or enzyme treatments [20,21,23]. Physical treatment, such as chopping, crushing, peeling, soaking, extruding, rolling, and steaming, can increase surface area, bulk density, and specific porosity to improve the digestive properties of the feed [21,62]. The use of solid-state fermentation (SSF) has shown promising results in improving the quality of the feedstuff [20]. SSF is a three-phase heterogeneous bioprocess fermentation through microbial cultivation [63]. In addition to that, enzyme treatments have been proposed to remove anti-nutritive factors in feed. Various enzymes, such as α-amylases, pectinases, lipases, tannases, xylanases, and phytases, have been studied to improve the quality of agricultural by-products [20]. Besides that, chemical treatments were also observed to change the by-products’ physical and chemical properties, further enhancing its utility. Untreated rice straws were noted to be high in lignin and silica, low in digestibility, protein, mineral, and vitamin content [64]. Chemical treatment, using 2.5% urea on rice straw, improved feed intake, digestibility, rumen fermentation, and microbial N synthesis efficiency in dairy cattle [65]. The intake and digestibility of untreated rice straw can also be improved through supplementation with other feedstuff, such as rice bran, cornmeal, urea, molasses, mineral blocks, and legume leaves [64]. Further research and development on the strategic manipulation in overcoming these obstacles to increase the usages of agricultural by-products would be key to safeguarding the feed supply for this growing industry.

Balancing the feeds’ energy and protein supply would enable its maximum utilization, while meeting the nutrient requirements of tropical dairy cattle breeds. Depending on the nutritional content, cattle diets can be catered for the different production stages, according to their nutritional needs. For example, to prevent abnormal rumen growth and attain good ruminal attrition, by-products with dietary fibers can be supplied to calves [21,66]. Many advances in estimating the nutrient availability of these feedstuffs have been recommended over the years [67,68,69]. Continuous research has been done to improve the utilization of agricultural by-products in the tropics, by studying their nutrient content [20,21,70]. The energy estimation can be improved when studies are refined for tropical conditions. For example, Detmann et al. [71] observed higher precision and accuracy in estimating the digestive fractions for equations in calculating energy when experiments are conditioned to the tropics. These models that incorporate tropical climate factors improve their goodness of fit when estimating the nutrient content of the feed available in the region.

Besides that, breaking down protein components in ration balancing would also improve protein supply. Metabolizable protein, for example, consists of microbial crude protein from ruminal degradable crude protein (RDP), rumen undegradable crude protein (RUP), and endogenous crude protein (ECP) [67,69]. Maximizing rumen degradable protein would increase microbial protein supply to the host, providing sufficient protein supply. The measure of RDP depends on anticipated or predicted microbial protein, whereby an increase in RDP supply would also increase the supply of microbial protein to the host [62]. Furthermore, microbial crude protein consists of 75% of the total protein supply of feed, which is turned into amino acids [62,72].

An accurate estimate of the agricultural by-products’ energy and protein are crucial to determining the optimal feed inclusion rates. Ultimately, the supply of intestinal protein or glucogenic nutrients, whether through propionic acid secretion or post-ruminal glucose infusion, influences milk lactose and protein in the milk. The nutrient is supplied in the form of amino acids (AA), glucose, acetate, and b-hydroxybutyrate (BHBA), which is supplied through the feed [73]. Therefore, a curvilinear increase in milk yield, protein, and lactose can be observed with an increased supply of energy and protein in the diet [73,74,75].

### 3.2. Economic Benefits through Strategic Use of Agricultural by-Products

Rising feed costs are a paramount concern for dairy farming in the tropics. Poor availability of quality feed material and higher feed cost may lead to poorer feeding value to livestock. The escalating feed cost may impact dairy milk yield, especially for small- to mid-scale farmers in this region. On average, feed cost can reach up to 43% of the total milk production cost, and even higher for countries dependent on the importation of feed, such as maize and soybean [18]. In addition, the use of maize in the US, sugarcane in Brazil, and vegetable oil and cereals in the EU (for the biodiesel and ethanol industries) has led to the rising concern of global feed price and shortages [1]. The expansion of the biodiesel and ethanol industry would mainly affect dairy farms reliant on grain as an energy supply for production. Furthermore, during a drought, feed prices may escalate due to the reduced availability of feed resources. In South Africa, agricultural yield declined by 8.4% in 2014, and feed prices soared 117% as drought conditions worsened from 2013 to 2016 [19]. Drought was also observed to affect pasture quality in the region, limiting the availability of feed resources for dairy farming and affecting milk production [7,19].

Feeding levels need to be pared with the genetic potential of the crossbred, though, in most instances, it will lead to increased feed cost [76]. Improving feed efficiency of available regional feed resources continues to be an important area of study to reduce the escalating cost of feed, while improving dairy farming profitability [18,77]. Agricultural residues could potentially be a cheaper alternative to provide sufficient nutrients for dairy production needs [20]. Carvalho et al. [78] postulated that the addition of up to 15% solvent extracted palm kernel meal had no adverse effect on the milk yield but reduced 4.2% of feed cost. Another study observed that cottonseed could replace soybean meal in rice straw and cassava-based diet and improved the dairy farmers’ income by between 10.5 to 13.7% [79]. Rice straw, fed with 50% fermented by-products of bagasse, pineapple peel, corn cob, corn stover, and vinasse, could significantly increase milk yield from 11.2 to 15.7 kg, as well as the short- and long-chain fatty acid contents, compared to cows fed solely with rice straw [80]. Furthermore, Pang et al. [81] concluded that replacing agro-industrial by-products with conventional concentrates had minimal impact on milk yield.

Broadening feed resources offers farmers alternatives to reduce the cost of feed, without severely impacting production levels. Strategic usage of agricultural by-products was seen as a coping mechanism used by farmers with feed scarcity and escalating feed cost associated with the tropical dry season [82]. During these conditions, some examples of the non-traditional feedstuff that can be used are by-products from oil palm production (oil palm press fiber), single-cell proteins, feed materials derived from agro-industrial of plant origin, poor-quality cellulosic roughages from farm residues and residues from the processing of sugar, cereal grains, as well as fruits and vegetables [20,21]. The indigenous cattle (Zebu) are also found to have higher efficiency in the digestion of low-quality forage rations [24]. Capitalizing on the genetic potential of the indigenous breeds in crossbreeding programs would allow the dairy cow to withstand drought periods, through better utilization of regional feed resources, particularly with lower nutritive values of non-conventional agricultural by-products. The ability of the tropical crossbreed to digest agricultural by-products more efficiently could be an area worth exploring, in order to capitalize on their economic merits.

Cost of production remains an essential factor for sustainable income to maintain or expand their dairying operation. Since milk is a commodity, milk price fluctuations, due to supply and demand, often poses a risk to many smallholders [9]. Cost of production is also related to the scale of the farm production, whereby most small-scale farms have a lower cost of production, due to lower output and integration with other agricultural activities [18]. The cost of milk in most tropical countries, such as Africa, Asia, and South America, had up to 25% lesser milk production cost than most temperate countries, except Brazil and China. One of the reasons observed was due to the usage of crop residue from their farm, resulting in a lower input system [18]. Therefore, the integration of agricultural activity with dairy farming would also potentially be a method for efficient land usage and to reduce the cost of feed with available farm residues for feed or bedding.

## 4. Heat Stress Impact and Mitigation

Heat plays a significant role in the nutrition, physiology of growth, and production in animals in the tropics. The environmental condition may be one of the main deterring factors in tropical dairy farming. The combination of high temperature and humidity in the tropics has been known to have adverse effects on the performance of dairy cattle breeds in the tropics [13]. The temperature humidity index (THI) is one of the measures of detecting heat stress. It was reported that the milk yield, fat, and protein were suppressed by 0.2, 0.012, and 0.009 kg, respectively, with every unit increase of the threshold at 72 [83]. Another study demonstrated that every increase per unit of THI decreases milk yield and DMI by 0.88 and 0.85 kg, respectively, in Holstein cattle above its threshold [22]. Heat stress effects were more prominent in Holstein crossbred housed in the non-cooled barn, with reduced DMI and milk yield observed [84]. The drop in milk yield during heat stress is a result of multiple compounded factors. Rhoads et al. [85] postulated that only 35% of the drop in milk yield was contributed from depressed DMI or nutrient supply, where the balance could have been contributed from postabsorptive metabolism and nutrient partitioning. Nevertheless, the increasing impact of climate change necessitates the need for resilient tropical crossbreds that can withstand harsher environments [31].

Diet reformulation has been used as a strategy to mitigate the effect of heat stress. Some of the nutritional effects of heat stress include a reduction of DMI, higher nutrient requirements, and nutrient wastages [22]. Reducing forage and increasing concentrate supplementation will reduce rumen retention time, while increasing energy supply [62,86,87,88]. The increase in concentrate will increase DMI, while reducing metabolic heat produced, due to fiber digestion [88]. Another method is through fat supplementation in feed to provide additional energy in the ration with minimal effect in heat increment [88]. Besides that, dietary protein degradability was also found to affect milk yield under heat stress conditions. Higher RUP, fed during hot weather, is observed to maintain DMI and improve milk yield [22]. Metabolic utilization for crude protein is higher, compared to starch or fat. This may be due to a higher protein turnover and the process of urea synthesis, which requires the deamination of the excess of amino acids (AAs) [88]. Supplementation with Lysin has also been observed to improve milk yield under heat stress conditions [89].

Energy requirement corrections were also seen as a method to improve production under heat stress conditions. Studies have shown that metabolizable energy for maintenance (Me_m_) of tropical crossbreds is lower than most temperate breeds. Oliveira [43] found that the heterosis breed (*Bos taurus × Bos indicus*) had 26% lower ME_m_ than temperate zone breed cattle. According to its severity, an estimated increase of 7 to 25% of maintenance energy requirements would be anticipated in heat stress conditions [62]. The increase in energy requirements is mainly due to increases in tissue metabolic rate, in order to dissipate heat [90,91]. NRC [92] suggested an equation to adjust requirements at 20 °C to the ambient temperature that changes the maintenance requirement by 0.0007 Mcal/kg SBW^0.75^ when exceeded. Fox and Tylutki [93] adjusted the maintenance requirement of dairy cattle in heat stress via environmental factors, such as ambient temperature, relative humidity, radiant energy, and wind speed. Another study found that the energy requirement needed during heat stress was modeled by predicting the animal’s surface area for the Holstein breed cow [94]. This is primarily because the capability of heat loss and tolerance to heat was observed to be due to the difference in skin morphology, related to its sweat glands [17]. Cooling management using sprinkler systems was found to be highly effective in preventing heat stress. Berman [13] noted that maximum evaporative losses were observed on the wetted hair coat. In addition, lowering air temperature and increasing air velocity or wind speed through improved ventilation were also critical management strategies to reduce the risk of heat stress [95]. Therefore, some minimal ventilative cooling systems in place would aid in improving heat stress management. Further studies can be done to evaluate the ability of tropical crossbreds to withstand the tropical climate, particularly the aspect of energy correction during heat stress.

Metabolic heat is also another main factor that causes heat stress in dairy cows [22]. According to their productive capability, lactating cows produce 27–48% more heat than non-lactating cows [96]. Therefore, high-performing temperate breeds are even more likely to suffer from heat stress, except for sufficient cooling mechanisms in place [94]. Although temperate and tropical crossbred metabolic heat differences could be due to their genetic attributes, their full extent has not been fully understood. Carabaño et al. [97] argued that selection for higher milking production traits might reduce heat stress tolerance in the dairy cow. More recently, genomic advances have paved the way for better selection for breeding [9]. For example, Dikmen et al. [98] identified a gene for heat tolerance (SLICK gene) that improves heat tolerance in Holstein cows. Technologies such as this would enable the selection of more superior performing tropical crossbreds that are more tolerant to tropical conditions.

## 5. Climate Change and Optimization of Dairy Production for Sustainable Farming in the Tropics

**Figure 1:**
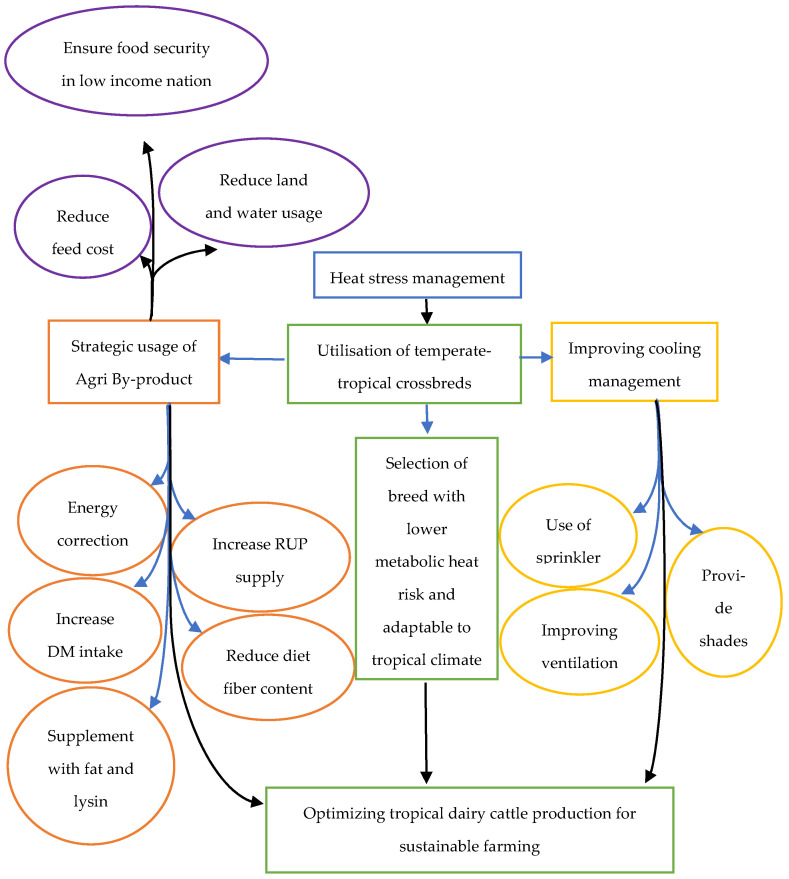
Optimizing of dairy cattle production for sustainable farming, to address heat stress in the tropic.

Minimalizing the carbon footprint of the dairy sector is an important step towards sustainable milk production, especially with the expansion of dairy farming in the tropics. Although greenhouse gas emissions (GHG) from dairy farming are considered minimal, compared to fossil burning and other more prominent contributors, this impact could be further reduced. GHG from dairy production was estimated at 4% of global GHG emissions, linked to milk production, processing, transportation, and culling for meat purposes. The highest GHG emissions expressed per kilogram of fat- and protein-corrected milk (FPCM) were observed in developing countries, especially in the tropical regions, such as sub-Saharan Africa, South Asia, North Africa, and the Near East [99]. The FAO report observed lower carbon emission in temperate countries, which may be attributed to higher feed efficiency through improved genetic selection, nutrition, and management [77,99]. Thornton and Herrero [100] emphasized reducing the number of animals by increasing the productivity of the dairy cattle breeds, as well as improving feed efficiency and digestibility, as strategies to reduce GHG emission. In addition, agroforestry can be adopted to improve carbon sequestration. For example, replacing *Leucaena leucocephala* as part of the basal diet could also be a valuable protein source for dairy cattle [100]. In India, field burning of crop residue contributed up to over 1% of the Indian agriculture sector’s GHG emissions [101]. Anaerobic digestion of the crop residue as animal feed was regarded as a more feasible green solution, by producing lower GHG emissions than traditional open burning, land application, and biowaste composting [101,102]. However, in a review, Yanti and Yayota [23] cautioned that the higher fiber content in some agricultural by-products might lead to increased GHG emission, though these are arguably smaller than conventional methods of agricultural residual disposal. Optimizing tropical dairy cattle production, illustrated in Figure 1, is crucial to dampen the effect of heat stress, while promoting sustainable farming with the strategic usage of agricultural by-products.

Tropical regions are exposed to the highest solar radiation, and climate change may directly or indirectly affect livestock through lower rainfalls, increased drought effect on crops, diseases incidence, and lower production [103]. Changes in sea-level temperature and increased tropical precipitation would lead to the anticipated formation of tropical cyclones [104]. As a consequence of these natural disasters, animal plant feed and crops would be impacted. In addition, changes in CO_2_ in the atmosphere may alter the growth rate, water-soluble carbohydrate, and nitrogen content, leading to lesser nutrient availability to dairy animals [105,106]. As part of a collective action to reduce the impact of global warming, the Paris Agreement 2015 has set a target of holding temperature increase to below 2 °C and pursuing efforts to limit to 1.5 °C [107]. The increasing global temperature may further deplete feed resources. With every increasing degree of Celsius, wheat production is expected to reduce by 6.0%, rice by 3.2%, maize by 7.4%, and soybean by 3.1% [108]. The reduction in agricultural yield would naturally also impact the by-products to be used as feed. Besides that, Africa and Latin America were projected to have a 10% reduction in maize production by 2055, due to climate change [109]. However, future advances in biotechnology are expected to mitigate this by improving the quality and digestibility of available feed resources [29,109]. Water resources are also crucial for irrigation, in order to produce sufficient animal feed. It is predicted that the consistent increase in global temperature would lead to a migration of farming areas, in the future, to regions with more sustainable water supplies [9]. It is estimated that animal production requires roughly 500 billion cubic water for feed production alone, and a mere 2% is used for drinking purposes [110].

Computer models, suited to tropical regions, have been designed to allow more efficient management of feed, land, and water resources, as well as improving milk performances. Mathematical models have been used to visualize optimal dairy farming by simulating regional conditions to maximize output [111]. As discussed in this review, the model would depend on the various input, including feeding value, breed requirements, and environmental and economic needs. Constraints added, such as minimal cost of feeding or environmental effect, can be incorporated into the model to formulate diets that meet regional policy [112]. Computer modeling may efficiently balance diets, while concomitantly utilizing available regional agricultural by-products [113,114,115,116]. Precision or accurate feeding allows minimal excretion of harmful substances, such as greenhouse gasses, nitrogen, and phosphorus, into the environment [111]. In addition, studies that form predictive estimates of heat stress and milk loss in dairy cattle are crucial for prevention methods to be employed before its occurrences [25,117]. These tropical dairy models could serve as a decision support system for tropical farms that provide possible solutions, based on the boundaries set using current scientific knowledge for sustainable farming [111].

Optimizing dairy farming in the tropics needs to come hand-in-hand with the production cost. The utilization of tropical feed resources and improvement of selected dairy cattle breeds, suited for tropical conditions, should be implemented, in conjunction with each other. Due to the fact that tropical crossbreeds require a long-term breeding plan and execution, the stable crossbred will be an asset to tropical countries in the future, especially when scarcity of feed resources and global warming becomes a closer reality. By utilizing regional agricultural by-products, and other feed resources with improved feeding regimes, the production level of tropical crossbreds can be improved to achieve a targeted average production level of 4500 kg in the tropics by 2050 [9].

## 6. Conclusions

In conclusion, while the introduction of temperate–tropical crossbreds is crucial to enhance dairy production in the tropics, it is also important to model and explore the impacts of incorporating agriculture by-products into the cow’s diet. No doubt, the performance of tropical crossbreds can be further improved through genetic selection and enhancing the use of feed resources and their efficiencies. This would reduce the feed cost and improve income, while promoting the sustainable use of land and water resources to meet this growing demand for dairy products. As climate change becomes a growing concern that may challenge future feed resources and dairy production in the tropics, preventive measures should be taken for continual optimal production levels. Improvement of tropical crossbreds, utilization of agricultural by-products in ration balancing, nutrient requirement correction, and management efforts were seen among measures to dampen the effect of heat stress and improve tropical dairy production. This would prevent losses up to 0.955 kg/day milk yield, due to heat stress, typically when metabolic heat production is at its peak. The optimization of breed attributes and feeding efficiency, in tandem with environmental parameters, briefly highlighted in the article, could help to model and be utilized as a predictive and decision support system for farmers. These are crucial tools that will safeguard the relevance of dairy farming amid global warming and greater pressure for higher dairy production in the tropics.

## Data Availability

Not applicable.
